# Coronary Artery Disease Is Related to Methylation Disorders Caused by the c.1286A>C *MTHFR* Polymorphism and to Low Serum 5-MTHF and Folic Acid Concentrations—Preliminary Results

**DOI:** 10.3390/reports7010006

**Published:** 2024-01-17

**Authors:** Agnieszka Pietruszyńska-Reszetarska, Robert Pietruszyński, Ireneusz Majsterek, Tomasz Popławski, Maciej Skrzypek, Beata Kolesińska, Joanna Waśko, Joanna Kapusta, Cezary Watała, Robert Irzmański

**Affiliations:** 1 Department of Internal Medicine, Rehabilitation and Physical Medicine, Medical University of Lodz, 90-647 Lodz, Poland; joanna.kapusta@umed.lodz.pl (J.K.); robert.irzmanski@umed.lodz.pl (R.I.); 2 Cardiology Outpatient Clinic, Military Medical Academy Memorial Teaching Hospital, Medical University of Lodz—Central Veterans’ Hospital, 90-549 Lodz, Poland; robpiet-portal@wp.pl; 3 Department of Clinical Chemistry and Biochemistry, Medical University of Lodz, 92-215 Lodz, Poland; ireneusz.majsterek@umed.lodz.pl (I.M.); tomasz.poplawski@umed.lodz.pl (T.P.); maciej.skrzypek@umed.lodz.pl (M.S.); 4 Institute of Organic Chemistry, Faculty of Chemistry, Lodz University of Technology, 90-924 Lodz, Poland; beata.kolesinska@p.lodz.pl (B.K.); joanna.wasko@p.lodz.pl (J.W.); 5 Department of Hemostasis and Hemostatic Disorders, Medical University of Lodz, 92-215 Lodz, Poland; cezary.watala@umed.lodz.pl

**Keywords:** methylation, 5-MTHF, folic acid, gene polymorphism, coronary artery disease, biomarker

## Abstract

Background: Single nucleotide polymorphisms in gene encoding is the key enzyme in the folates pathway, methyltetrahydrofolate reductase (MTHFR), which causes methylation disorders associated with coronary artery disease (CAD). We evaluated associations between methylation disorders caused by *MTHFR* gene polymorphisms and the blood folate concentrations (folic acid, 5-MTHF) in CAD patients. Methods: Study group: 34 patients with CAD confirmed by invasive coronary angiography (ICA). Controls: 14 patients without CAD symptoms or significant coronary artery stenosis, based on ICA or multislice computed tomography (MSCT) with coronary artery calcification (CAC) scoring. Real-time PCR genotyping was assessed using TaqMan™ probes. Folic acid and 5-MTHF concentrations in blood serum were determined using Liquid Chromatography-Mass Spectrometry (LC-MS). Results: The c.[1286A>C];[1286A>C] *MTHFR* polymorphism occurred significantly more often in (CAD^+^) patients compared to the (CAD^−^) cohort and to the selected general European “CEU_GENO_PANEL” population sample. The concentration of 5-MTHF and folic acid in subgroups of CAD^+^ patients with methylation disorders categorized by genotypes and CAD presence (CAD^+^) was always lower in CAD^+^ subgroups compared to non-CAD individuals (CAD^−^). Conclusions: Further studies on a larger scale are needed to implicate the homozygous c.1286A>C *MTHFR* variant as CAD genetic marker and the 5-MTHF as CAD biomarker. Identification of high CAD risk using genetic and phenotypic tests can contribute to personalized therapy using an active (methylated) form of folic acid (5-MTHF) in CAD patients with *MTHFR* polymorphisms.

## 1. Introduction

CAD, which is the major representative of cardiovascular diseases, is the main cause of mortality in developed countries [1], leading to premature death and disability in humans [2]. In 2010, CAD was responsible for 19 million deaths in developing countries [3]. Despite multiple COVID-19 pandemic deaths, cardiovascular diseases remained the leading cause of mortality in Poland [4].

CAD is caused by the narrowing or occlusion of the epicardial arteries in the heart by atherosclerotic lesions, which leads to coronary flow impairment. The management of a patient with CAD depends on the degree of coronary artery stenosis, diagnostic test results, and the severity of clinical symptoms.

Although significant progress in terms of non-invasive and invasive methods of diagnostics, including multislice computed tomography (MSCT) with coronary artery calcification scoring (CAC scoring), coronary CT angiography (CCTA), and invasive coronary angiography (ICA), as well as methods of CAD treatment, there is still a need to search for new genetic and phenotypic CAD markers for early detection and prevention of CAD.

There are a number of risk factors contributing to the development of CAD, including genetic, psychosocial, socioeconomic, and environmental ones, as well as those linked to ethnicity, body composition, frailty, or family history [5]. Genetic factors can contribute as much as 40–60% to the disease [6]. The etiology of the disease is complex, and the variable phenotype is the result of interactions between multiple genes and environmental factors [7]. Inheritance of CAD can be either monogenic or polygenic [8]. Monogenic diseases are rare and usually revealed in early childhood, whereas the most common diseases, such as CAD, are polygenic inherited. Genetic polymorphisms associated with CAD confirm not only the importance of established risk factors but also the existence of many new causal pathways that are expected to improve our understanding of CAD fundamentals [8,9].

The term polymorphism, which refers to different allelic forms of DNA in a population where the minimum frequency of the rarer allele is 1%, may involve a single or repeated nucleotide sequence motif, as well as the number of copies of the DNA fragment. Single nucleotide polymorphisms (SNPs) in the DNA are detected by sequencing. Research in this area focuses on genome-wide association studies (GWAS) [6]. The mechanisms responsible for the inflammation, endothelial function, platelets function, lipid metabolism, development of thrombosis, insulin sensitivity, and blood pressure regulation are being taken into account. For example, the anti-inflammatory adipokine, vaspin, secreted by adipose tissue, has been recently proven to play a role in CAD development [10]. However, the pathomechanism of the majority of novel polymorphisms in CAD development requires further investigation [8], and the use of genetic markers is not recommended. The only exception is in monogenic inherited cardiovascular diseases caused by the LDLR mutation [11].

**Figure 1:**
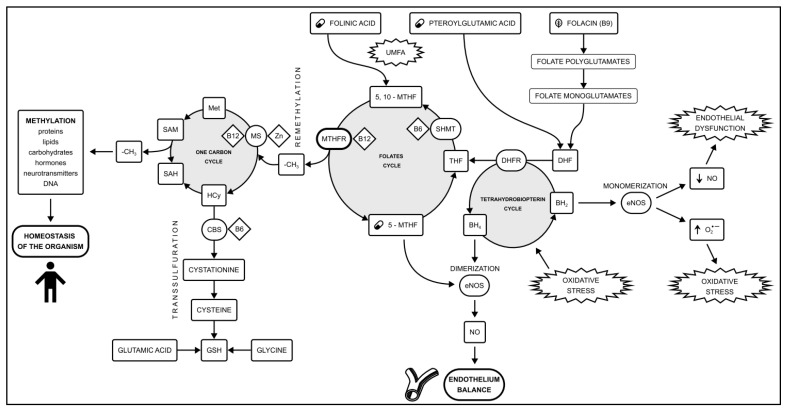
The methylation pathway is formed by the one-carbon cycle and the folate cycle. An additional role is played by the transsulfuration pathway and the tetrahydrobiopterin cycle. Methylation disorders caused by the *MTHFR* polymorphisms play a role in the development of vascular endothelial dysfunction. The 5-MTHF availability plays a key role in the amount of circulating NO. Shifting the balance between NO production and oxidative stress in endothelial cells is a key step in CAD development [22,23,24]. Abbreviations used: (O_2_^•−^) Superoxide anion radical; 5,10-MTHF—5,10-methylenetetrahydrofolate; 5-MTHF—methylated folic acid, levomefolic acid; BH2—Dihydrobiopterin; BH4—Tetrahydrobiopterin; CAD—coronary artery disease; CBS—cystathionine β-synthase; DHF—dihydrofolate; DHFR—dihydrofolate reductase; eNOS—endothelial nitric oxide synthase; GSH—reduced glutathione; Hcy—Homocysteine; Met—Methionine; MS—Methionine synthase; MTHFR—methyltetrahydrofolate reductase; NO—nitric oxide; SAH—S-adenosylhomocysteine; SAM—S-adenosylmethionine; SHMT—serine hydroxymethyltransferase; THF—tetrahydrofolate; UMFA—Unmetabolized Folic Acid syndrome.

Another approach is the candidate gene method, focusing on the relationship between genetic variation and phenotype [12]. Based on the pathophysiology, we can assume which genes are important in the development of the disease, but more importantly, whether they can be a point of reference for pharmacotherapy. One example of this point of view is the research into methylation disorders caused by the SNPs in the methyl tetrahydrofolate reductase (*MTHFR*) gene. Methylation is a process when a methyl group (-CH3) is transferred between donors and acceptors such as neurotransmitters, lipids, proteins, and DNA [13]. Methylation plays a key role in maintaining the homeostasis of the organism and supporting the function of endothelium [14] (Figure 1). Methylation disorders have been linked, apart from CAD, with various disorders such as Down syndrome, multiple sclerosis, ischemic stroke, intrauterine death, obstetric failures, and lung and hematopoietic system tumors.

MTHFR, which is a key enzyme in the folate pathway. Naturally occurring folates are folacin, found in vegetables, and methyltetrahydrofolate (5-MTHF), representing an active form of folic acid in the blood. MTHFR catalyzes the conversion of 5,10-methylenetetrahydrofolate to the 5-MTHF. 5-MTHF’s availability plays a key role in the amount of nitric oxide (NO) [15]. Genetic variation in *MTHFR* polymorphism may influence the concentration of folates and, consequently, their biological functions in the process of methylation. Shifting the balance between NO production and oxidative stress leads to endothelial dysfunction (ED), which is a key step in CAD development [16]. The most common *MTHFR* SNPs are c.665C>T (rs1801133) and c.1286A>C (rs1801131). These polymorphisms can occur as a heterozygous genotype (polymorphism in only one allele), homozygous (polymorphism in both alleles), and in the form of complex heterozygotes (one of the above-mentioned polymorphisms in one allele and the other in the other allele). According to the Genome Aggregation Database (gnomAD), in the general European population, the c.665C>T polymorphic allele affects 30.85% of the population, and the c.1286A>C allele affects 28.58% of the population [17]. The presence of the *MTHFR* polymorphism results in a loss of 40 to 70% of enzyme function for variant c.665C>T and 30 to 50% for variant c.1298A>C [18].

*MTHFR* polymorphisms are associated with conditions such as cardiovascular diseases, cancer, neurological diseases, diabetes, and psoriasis. In the past, they have been linked to thromboembolism; however, the determination of *MTHFR* polymorphisms in thrombophilia screening is currently not recommended by scientific societies [18,19].

Currently, CAD prevention methods center around making changes to a lifestyle or pharmacotherapy for lipid disorders and hypertension. Research on the impact of methylation disorders on the development of cardiovascular diseases showed conflicting conclusions and was discontinued [20]. According to the standards regarding folic acid and B vitamins (B6, B12) and supplementation, no beneficial effects have been demonstrated [21].

We aimed to search for new genotypic and phenotypic biomarkers for the development of CAD. Since the folate serum concentrations depending on the genotype of CAD patients have not been investigated so far, analyzing these relationships is important to improve cardiovascular risk estimation. The rationale for our study was rooted in the hypothesis that patients with CAD have endothelial dysfunction caused by *MTHFR* polymorphisms. The objective was to examine whether the methylation disorders caused by the c.665C>T and c.1286A>C *MTHFR* polymorphisms occur more often in patients with confirmed CAD on the basis of ICA or MSCT, as well as whether a lower concentration of 5-MTHF in the blood of CAD cases.

This research may potentially lead to new directions in therapy, particularly the usage of 5-MTHF in CAD patients with methylation disorders caused by *MTHFR* gene polymorphisms.

## 2. Materials and Methods

### 2.1. Study Design and Population

All of the participants (Caucasian, recruited from February 2020 to October 2022) signed an informed consent. The Ethics Committee of the Medical University of Lodz (Poland) approved this case–control study. We followed the ethical guidelines of the Declaration of Helsinki. We used the cluster (in accordance with the outcomes of ICA/MSCT with CAC scoring) consecutive sampling algorithm (see below).

### 2.2. Inclusion/Exclusion Criteria

All participants underwent invasive coronary angiography (ICA) (elective or due to acute coronary syndrome (ACS) or multislice computed tomography (MSCT) with coronary artery calcification scoring (CAC scoring)).

Inclusion criteria: ≥50% stenosis of the left main coronary artery or ≥70% stenosis of one or more other major coronary arteries based on Quantitative Coronary Analysis (QCA) of the vessels: left anterior descending artery (LAD); circumflex branch of the left coronary artery (Cx); right coronary artery (RCA).

The control group included patients without CAD clinical symptoms nor significant coronary artery stenosis based on ICA or MSCT with CAC scoring.

Exclusion criteria: chronic inflammatory diseases, severe anemia, neoplastic diseases, immunosuppressive therapy, renal failure, thrombophilia, history of venous thromboembolism, familial hypercholesterolemia.

#### Blood Collection

The blood was collected into two tube syringes: 4.9 mL of whole blood with ethylenediaminetetraacetic acid (EDTA) for genetic analysis and 2.6 mL of serum isolated from another portion of whole blood with clotting activator/gel for 5-MTHF and folic acid analysis, both collected in the morning, after a 30 min rest from 213 patients.

### 2.3. Genetic Analysis

Genomic DNA was isolated from peripheral venous blood samples using the GeneMatrix Blood DNA purification Kit (EURx, Gdańsk, Poland) in line with the manufacturer’s protocol. DNA concentration and purity were determined spectrophotometrically by measuring the absorbance at 260 and 280 nm on a Synergy HT spectrophotometer (BioTek, Hong Kong, China). Identification of polymorphic variants of the *MTHFR* gene c.665C>T (the terms C677T or C665T are no longer recommended; rs1801133 according to the Single Nucleotide Polymorphism Database) and c.1286A>C (the terms A1298C or A1286c are no longer recommended; rs1801131) was performed using TaqMan^®^SNP and TaqMan Universal PCR MasterMix, No UNG (Applied Biosystems, Foster City, CA, USA) genotypic tests. A kit with primers and fluorescently labeled molecular probes was used for genotyping during real-time DNA polymerase chain reaction analysis. Markings were made in accordance with the manufacturer’s recommendations. The total volume of the PCR reaction was 20 μL, including 4 μL of 5×HOT FIREPol^®^Probe qPCR Mix (Solis, Tartu, Estonia), 1 μL of DNA (100 ng), 1 μL of TaqMan SNP primers and 14 μL of RNA-free water. The PCR conditions were as follows: polymerase activation (10 min, 95 °C), 30 cycles of denaturation (15 s, 95 °C) annealing/extension (60 s, 60 °C). Genotyping was performed in the Bio-Rad CFX96 system (BioRad, Hercules, CA, USA).

### 2.4. Folates Analysis

The Liquid Chromatography-Mass Spectrometry (LC-MS) method was used to determine the concentrations of folic acid and 5-MTHF in blood serum. LC-MS is a combined technique used in the qualitative and quantitative analysis of complex biological samples. Only the LC-MS tests are able to distinguish between different forms of folic acid: 5-MTHF, folic acid, and folinic acid, and they should be used in the assessment of folate status, unlike the mass-used tests based on fluorescence, evaluating the level of all folates containing the pteroyl ring, without differentiating them. The use of such tests does not reflect the level of the biologically active form of folic acid [25]. Tests detecting 5-MTHF are not commonly used despite the fact that only these tests enable the assessment of the concentration of a metabolically active methyl group donor. They also allow the actual reflection of the status of folic acid in *MTHFR* gene polymorphism subjects [26].

The LC-MS analysis was carried out using a DIONEX UltiMate 3000 liquid chromatograph equipped with a DAD (diode array detector) from Thermo Scientific (Waltham, MA, USA) coupled with a microOTOF-QIII mass spectrometer from Bruker (Billerica, MA, USA).

- Preparation of the sample for analysis: 1 mL of EtOH was added to the 0.5 mL of the sample, mixed together, and transferred to an Eppendorf (2 mL). Next, the sample was rinsed with an additional 0.5 mL of EtOH. Eppendorfs incubated at 37 °C for 20 min. After incubation, the probes were centrifuged. The supernatant was collected.
- LC analysis: gradient elution (component A—water and component B—acetonitrile, both solvents with the addition of 0.1% formic acid); reverse phase column by Kinetex C18 100 × 4.6 mm without thermostating, particle size—2.6 µm, pore size 100 Å; analysis program (B/A): 0–2 min 3/97, 2–31 min 95/5, 31–32 min 0/100, 32–33 min 0/100, 33–35 min 3/97, 35–37.5 min 3/97; analytical wavelengths of the DAD detector: 214, 220, 256 and 291 nm; sample injection volume per column—10 µL.
- MS analysis: electrospray ionization method, the flow rate of drying gas (nitrogen) 6.0 L/min, nebulizer pressure 2.4 Bar, capillary inlet temperature 250 °C, capillary voltage 4000 V; TOF (time of flight) detector. Solvents included in the eluent and used for sample preparation had the degree of purity required for LC-MS analysis. All obtained solutions were additionally centrifuged in order to remove possible impurities and residues of undissolved compounds.

The obtained chromatograms and MS spectra were processed using the DataAnalysis 4.2 program.

### 2.5. Statistical Analysis

Shapiro–Wilk test and Brown-Forsythe test were applied to verify, respectively, for the normality of data and the homogeneity of variance. Significance of differences between two independent groups in the cases when the assumptions of either data normality or variance homogeneity or both were violated, was estimated with Mann–Whitney’s U test or Kruskal–Wallis’ test. Otherwise, Student’s *t*-test for independent samples or simple one-way ANOVA, followed by the post hoc multiple comparison tests, were used. Multiple comparisons were checked post hoc using either Connover-Inman (nonparametric) or Tukey’s test (parametric). Data that did not meet the condition of normal distribution/variance homogeneity were analyzed with the Kruskal–Wallis test, using the classical approach, and with the use of bootstrap simulation (Monte Carlo model, 1,000,000 iterations). Multi-parametric logistic regression, with the adjustment for either age and gender or for age, gender, and folic acid/5-MTHF serum concentrations, was used to analyze the relationship between the variables. The V-fold cross evaluation was used as a validation method. The fit of the model was checked with the Hosmer-Lemeshow. Due to the relatively small sample size and the low statistical power of the estimated inferences in the majority of multivariate calculations, a resampling with replacement (bootstrap, 10,000 iterations) was used to minimize the risk that the observed probability demonstrating the revealed significant difference differences was encountered by pure chance. Statistical analysis was carried out using Statistica v. 12.5 and Resampling Stats Add-in for Excel v.4. *p* values less than 0.05 were considered statistically significant.

## 3. Results

### 3.1. Characteristics of the Groups

The preliminary analysis was made for the 48-patient group. We divided the subjects according to the presence of CAD (CAD^+^/^−^) and the *MTHFR* polymorphisms. The study group (CAD^+^) included 34 patients (31 males; 3 females). All patients in this group underwent ICA. The control group (CAD^−^) included 14 patients (11 males; 3 females) with no coronary stenosis based on ICA or MSCT with CAC score (Table 1).

### 3.2. Frequencies of the Genotypes

In the (CAD^+^) group, the frequency of genotypes c.[665C=];[665C=] (both wild-type alleles, no polymorphism); c.[665C>T];[665C=] (heterozygous c.665C>T) and c.[665C>T];[665C>T] (homozygous c.665C>T) were, respectively, 32.4%, 26.5%, 41.1% vs. 21.4%, 21.4%, 57.1% for (CAD^−^) (Table 2).

The C (“wild-type”) allele occurred in 45.6% of (CAD^+^) and in 32.1% of the (CAD^−^) group. The T (polymorphic) allele occurred in 54.4% of (CAD^+^) and in 67.9% of the (CAD^−^) group.

Genotypes c.[1286A=];[1286A=] (both wild-type alleles, no polymorphism), c.[1286A>C];[1286A=] (heterozygous c.1286A>C) and c.[1286A>C];[1286A>C] (homozygous c.1286A>C) occurred at, respectively, 8.8%, 35.3%, 55.9% of (CAD^+^) compared to 21.4%; 64.3%, 14.3% of (CAD^−^) group.

The A (“wild-type”) allele occurred in 26.5% of (CAD^+^) and 53.6% of (CAD^−^) groups. The C (polymorphic) allele occurred in 73.5% of (CAD^+^) and 46.4% of the (CAD^−^) group.

#### 3.2.1. Coronary Artery Disease Patients versus Controls Comparisons

We found that c.[1286A>C];[1286A>C] (homozygous c.1286A>C) *MTHFR* polymorphism was statistically significantly more frequent in (CAD^+^) compared to (CAD^−^) group. Comparisons were made using bootstrap-boosted logistic regression analysis (OR = 40.327; 95% CI 1.860–874.464; *p* = 0.019) (Table 2).

The c.[1286A>C];[1286A=] polymorphism (heterozygous c.1286A>C) was statistically significantly less frequent in (CAD^+^) compared to (CAD^−^) (OR = 0.039; 95% CI 0.002–0.687; *p* = 0.027). The c.[1286A=];[1286A=] genotype (both wild-type alleles) was more common in the (CAD^−^) group, but the changes were not statistically significant.

A significantly higher incidence of the C allele (polymorphic, occurring both in homozygous c.[1286A>C];[1286A>C] and heterozygous c.[1286A>C];[1286A=] genotypes) was observed in the (CAD^+^) group compared to (CAD^−^) (OR = 4.930; 95% CI 1.403–17.321; *p* = 0.009)

To conclude, there was no statistically significant relationship between the occurrence of *MTHFR* c.665C>T gene polymorphisms and CAD. Otherwise, we found such associations with respect to *MTHFR* c.1286A>C gene polymorphisms and CAD. In all the above relationships, the examined genotypes and alleles were independent of age, gender, serum folates, and serum 5-MTHF concentrations.

#### 3.2.2. Coronary Artery Disease Patients versus European Population Comparisons

We compared the frequency of homozygous c.1286A>C genotype in the (CAD^+^) group to the frequency in the European population “CEU_GENO_PANEL”, which consisted of 60 people (30 men, 30 women), selected for the statistical comparison from the NCBI (National Center for Biotechnology Information, USA) database database [27].

Genotypes c.[1286A=];[1286A=] (both wild-type alleles, no polymorphism), c.[1286A>C];[1286A=] (heterozygous c.1286A>C) and c.[1286A>C];[1286A>C] (homozygous c.1286A>C) occurred at, respectively, 41.67%, 45.00% and 13.33% of CEU_GENO_PANEL group. The A (“wild”) allele occurred in 64.17%, the C (polymorphic) allele in 35.83%.

We found that c.[1286A>C];[1286A>C] (homozygous c.1286A>C) *MTHFR* polymorphism was statistically significantly more frequent, whereas the allele A—statistically less frequent in the (CAD^+^) group than in the European population. Comparisons were made using bootstrap-boosted logistic regression analysis (OR = 8.821, 95% CI 3.416–22.782, *p* < 0.0001 and OR = 0.201, 95% CI 0.104–0.387, *p* < 0.0001, respectively, for the gene and for the allele).

Overall, there was a statistically higher incidence of c.1286A>C *MTHFR* homozygotes and polymorphic “C” allele in CAD patients than in the selected general European population sample.

### 3.3. Folates Concentrations

#### 3.3.1. Folates Concentrations in Patients with MTHFR Polymorphism

We observed that in c.[665C=];[665C=] patients (“wild type”), the folic acid concentration in the serum was the highest of the three possible c.665 genotypes (1.95 μg/L). In c.[665C>T];[665C=] (heterozygous) patients, the mean serum folic acid concentration was lower than in the wild type (1.90 μg/L). In c.[665C>T];[665C>T] (homozygous) patients, the mean serum folic acid concentration was lower than in the wild type (1.90 μg/L) (Table 3).

In conclusion, the serum folate levels according to the genotype, without taking the presence or absence of CAD into account, showed no significant differences between the groups.

#### 3.3.2. Folates Concentrations According to the MTHFR Genotype and the Occurrence of Coronary Artery Disease

We compared the serum folate concentration according to the presence and absence of CAD in matching genotype groups.

**Figure 2:**
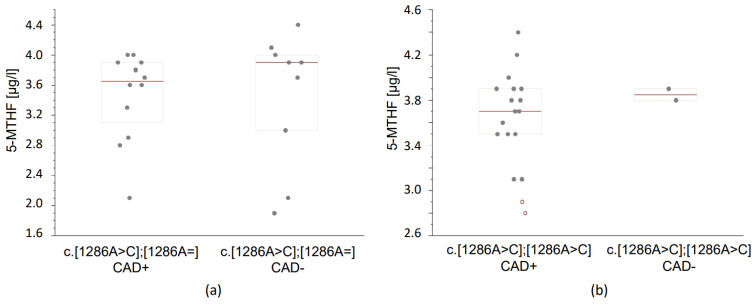
Concentrations of 5-MTHF in blood serum of CAD^+^ and CAD^−^ patients. Data, given in μg/L, are presented as medians (thick horizontal lines) and quartile ranges (boxes); raw estimates are also given. Values were evaluated using the LC-MS method. (**a**) Concentrations of 5-MTHF were lower in c.1286A>C heterozygous (CAD^+^) patients compared to (CAD^−^) ones (Mann–Whitney U, *p* = 0.269); (**b**) Concentrations of 5-MTHF were lower in c.1286A>C homozygous (CAD^+^) patients compared to (CAD^−^) individuals (Mann–Whitney U, *p* = 0.286).

In the group of c.[1286A>C];[1286A=] (heterozygous c.1286A>C) patients and CAD, a lower concentration of folic acid was observed (1.8 μg/L) compared to the matching group without CAD (2.0 μg/L). In the group of c.[1286A>C];[1286A=] (heterozygous c.1286A>C) patients and CAD, a lower concentration of 5-MTHF (3.65 μg/L) was observed compared to the matching group without CAD (3.90 μg/L) (Figure 2a). In the group of patients with c.[1286A>C];[1286A>C] (homozygous c.1286A>C) and CAD, a lower concentration of 5-MTHF (3.7 μg/L) was observed compared to the matching group without CAD (3.85 μg/L) (Figure 2b). However, the observed differences between the groups did not reach significance (Table 4).

A comparison of the (CAD^+^) versus (CAD^−^) patients with matching c.665C>T genotypes did not show statistically lower concentrations of folic acid and 5 MTHF in the (CAD^+^) group. However, we observed that in the c.665 homozygous patients with CAD, serum folic acid concentration was lower (1.9 μg/L) compared to the matching (CAD^−^) group (1.95 μg/L). The concentration of 5-MTHF was lower in c.665 homozygous patients with CAD (3.7 μg/L) compared to the matching (CAD^−^) group (3.8 μg/L).

Overall, the concentration of 5-MTHF and folic acid was lower in CAD^+^ and c.665C>T *MTHFR* homozygotes compared to non-CAD individuals (CAD^−^).

Moreover, the concentration of 5-MTHF was always lower in CAD^+^ patients with methylation disorders due to c.1286A>C *MTHFR* polymorphisms than in the CAD^−^ patients.

## 4. Discussion

This paper presents the effect of methylation disorders caused by *MTHFR* polymorphisms on folic acid and 5-MTHF metabolism in CAD patients. We showed a significantly higher incidence of c.1286A>C homozygous *MTHFR* polymorphism in CAD patients confirmed using ICA compared to controls without significant coronary artery stenosis based on ICA or MSCT with CAC scoring. In addition, we found out that c.1286A>C homozygous variant is significantly more frequent in CAD patients compared to the general European CEU_GENO_PANEL population sample. We showed a lower occurrence of the c.1286A>C heterozygote in CAD patients but a higher occurrence of the C allele in the CAD group.

Additionally, the present research demonstrates lower concentrations of 5-MTHF and folic acid in the subgroup of CAD and c.665C>T *MTHFR* patients compared to matching non-CAD individuals. This study reveals that the concentration of 5-MTHF was always lower in CAD patients with methylation disorders due to c.1286A>C *MTHFR* polymorphisms.

Impaired methylation is significant in the pathogenesis of cardiovascular diseases [28]. Reduced methylation ability is determined using *MTHFR* polymorphisms and affects about 30% of the European and 40% of the Polish population [29]. Methylation disorders are associated with CAD [30]. Significant associations between the c.1286A>C *MTHFR* polymorphisms and CAD occurrence were proved [31,32,33]. The incidence of c.665C>T polymorphism obtained in this study did not differ significantly between groups. However, the T allele of the c.665C>T polymorphism was found to be a risk factor for CAD [30].

Our results, showing that 5-MTHF and folic acid concentrations were lower in heterozygotes and in homozygotes with methylation disorders caused by *MTHFR* c.665C>T and c.1286A>C polymorphisms, were consistent with outcomes regarding folic acid provided by other researchers [34,35]. However, in a whole-genome sequencing study it appeared that the other gene located on the same chromosome as *MTHFR* was responsible for folic acid concentration [36].

There are hitherto no publications regarding 5-MTHF serum concentrations depending on the CAD patients’ genotype; however, an association of a low 5-MTHF and other diseases with the same risk factors as CAD, including obesity [37], smoking [38] and hypertension [39], is being investigated. An increased risk of death from any cause was associated with low 5-MTHF [40]. A lower 5-MTHF concentration was found in CAD patients [41].

When it comes to the methodology, tests detecting 5-MTHF are not common, despite the fact that they enable direct assessment of active methyl group donors and reflect the folate status properly. Measurements using fluorescence-based tests assess the level of all folates containing a pteroyl ring without differentiating them. Tests based on LC-MS are able to distinguish between 5-MTHF and folic acid, but their usage is limited to scientific research [26]. In the present study, folic acid and 5-MTHF concentrations were determined using LC-MS in serum. The lack of statistical significance regarding folates in our research may result from the limited size of groups categorized by the genotypes. It is reasonable to expand the size of the study to confirm our findings.

Concerning the inclusion/exclusion criteria, there are many papers considering only the absence of angina or ischemic changes in the ECG as an exclusion criterion [42]. Despite the relatively small sample, all of the patients included have undergone ICA or MSCT with CAC Scoring to exclude the asymptomatic patients with identifiable atherosclerotic lesions in the coronary arteries.

### Practical Aspects of Results and Future Directions of Research

In cardiovascular disease prevention, a folate-rich diet (>200 g/day of fruit and vegetables) is recommended [21]. The reference intake of folic acid should be 200 μg per day—mainly from green vegetables. The current recommendations do not diversify the patients concerning genotype, nor do they regard the additional use of folates depending on *MTHFR* polymorphisms in CAD patients. The optimal level of folates may be particularly important for the health of people with methylation disorders.

Contrarily, in central nervous system defects prevention in pregnancy planning, 400 μg of folic acid or its active metabolites a day is recommended. High doses of folic acid (up to 4–5 mg daily) are recommended for women who have given birth to children with such a defect [43]. However, the form of folic acid (normal or methylated) does not depend on the genotype.

High doses of folic acid (>400 ug per day) are potentially harmful. Supplementation with synthetic folic acid can lead to Unmetabolized Folic Acid (UMFA) syndrome [44]. Synthetic folic acid may activate cell division in the vascular smooth muscles, increasing proliferation. After coronary artery revascularization, in patients in whom high (>1 mg) doses of folic acid were used, restenosis in stents occurred significantly more often [45]. Mandatory food fortification with synthetic folic acid has been implemented in the prevention of neural tube defects in over 80 countries [46]. It is reasonable to ask whether people with methylation disorders caused by the *MTHFR* polymorphism will benefit from such a procedure.

We conclude that it should be predominantly the bioavailability of folic acid and the patient’s genotype that are taken into consideration first, in contrast to the current approach that focuses on increasing the dosage of folic acid.

## 5. Conclusions

Our study assessed folic acid and its active form, methyltetrahydrofolate (5-MTHF) concentrations in blood serum, depending on the *MTHFR* genotype in CAD patients. The results of this study showed that in patients with angiographically confirmed CAD, the c.[1286A>C],[1286A>C] *MTHFR* polymorphism (homozygous c.1286A>C) occurred significantly more often in CAD patients as well as compared to the European “CEU_GENO_PANEL” population. Despite the relatively small group studied, the observed relationship is statistically significant. Genetic predisposition (homozygous c.1286A>C) contributed to the lower levels of 5-MTHF in this group of patients.

Further studies on a larger scale are needed to implicate the homozygous c.1286A>C *MTHFR* variant as a CAD genetic marker and the 5-MTHF as a CAD biomarker.

Identification of high CAD risk using genetic and phenotypic tests can contribute in the future to personalized therapy using an active (methylated) form of folic acid (5-MTHF) in CAD patients with *MTHFR* polymorphisms.

## Figures and Tables

**Table 1:** Demographic and clinical characteristics of the study groups depending on the occurrence of coronary artery disease.

Parameters	CAD^+^ (*n* = 34)	CAD^−^ (*n* = 14)	*p*-Value
Sex (% males)	31/34 (91.2%)	11/14 (78.6%)	0.339
Mean age (years)	52 (47–58)	59 (56–61)	0.010 ^#^
Mean age at CAD diagnosis	47.1 + 5.6	N/A	
Total cholesterol (mg/dL)	195.5 + 48.2	179.3 + 39.9	0.366
HDL Cholesterol (mg/dL)	47.1 + 12.1	56.2 + 19.4	0.295
LDL Cholesterol (mg/dL)	116.8 + 38.8	101.7 + 31.9	0.096
Triglycerides (mg/dL)	148 (93–206)	81 (71–127)	0.015 ^#^
non-HDL Cholesterol (mg/dL)	148.4 + 46.8	123.3 + 29.6	0.136
LVEF < 50% (TTE)	15/33 (45.5%)	4/11 (36.4%)	0.731
Hypertension	20/34 (58.8%)	4/14 (28.6%)	0.111
Obesity (BMI > 30 kg/m^2^)	11/34 (32.4%)	3/14 (21.4%)	0.510
Dyslipidemia	19/34 (55.9%)	4/14 (28.6%)	0.193
Diabetes	5/34 (14.7%)	2/14 (14.3%)	0.998
Family history (+)	8/34 (23.5%)	1/14 (7.1%)	0.410
Nicotinism	19/34 (55.9%)	2/14 (14.3%)	0.020
Past ACS	18/34 (52.9%)	N/A	
Past PCI	30/34 (88.2%)	N/A	
Past CABG	7/34 (20.6%)	N/A	
ASA	33/33 (97.1%)	1/13 (7.7%)	<0.0001
DAPT	29/33 (87.9%)	0/13 (0.0%)	<0.0001
ACEI/ARB	28/33 (84.8%)	8/13 (61.5%)	0.117
β-blockers	31/33 (93.9%)	7/13 (53.8%)	0.004
Ca-blockers	1/33 (2.9%)	3/13 (21.4%)	0.062
Diuretics	6/33 (18.2%)	3/13 (23.1%)	0.698
Statins	32/33 (97.0%)	1/13 (7.7%)	<0.0001

Data for continuous variables are presented as unadjusted means ± SD or medians and quartile ranges. Discrete data are presented as absolute values (fractions, %). The significance of differences between the groups was assessed using the Student’s *t*-test or Mann–Whitney U test (^#^). Abbreviations: ACEI/ARB—angiotensin-converting-enzyme inhibitors/Angiotensin II receptor blockers; ACS—Acute Coronary Syndrome; ASA—Acetylsalicylic Acid; β-blockers—Beta-adrenergic blocking agents; Ca-blockers—Calcium channel blockers; CABG—coronary artery bypass grafting; CAD—Coronary Artery Disease; DAPT—dual antiplatelet therapy; HDL—high-density lipoprotein; LDL—low-density lipoprotein; LVEF—left ventricle ejection fraction; N/A—not applicable; PCI—percutaneous coronary intervention; TTE—transthoracic echocardiogram.

**Table 2:** Distribution of genotypes and alleles of the studied single-nucleotide *MTHFR* polymorphisms in groups of patients with coronary artery disease (CAD^+^) and controls without identified stenoses in the coronary arteries (CAD^−^).

Genotype/Allele	CAD^+^ (*n* = 34)		CAD^−^ (*n* = 14)		Crude OR (95% CI)	*p*	Adjusted OR (95% CI) *	*p*	Adjusted OR (95% CI) ^#^	*p*
	Number	Frequency	Number	Frequency						
	*MTHFR* c.665C>T									
c.[665C=];[665C=]	11	0.324	3	0.214	1.754 (0.405–7.588) ^&^ 1.844 (0.375–9.065)	0.452 0.451	2.001 (0.370–10.823) ^&^ 2.149 (0.328–14.080)	0.420 0.425	2.144 (0.385–11.929) ^&^ 2.748 (0.306–24.668)	0.384 0.367
c.[665C>T];[665C=]	9	0.265	3	0.214	1.320 (0.298–5.838) ^&^ 1.322 (0.265–6.598)	0.714 0.734	1.434 (0.267–7.697) ^&^ 1.397 (0.195–10.016)	0.674 0.740	1.347 (0.246–7.378) ^&^ 1.536 (0.158–14.933)	0.731 0.712
c.[665C>T];[665C>T]	14	0.411	8	0.571	0.525 (0.149–1.850) ^&^ 0.507 (0.133–1.933)	0.316 0.320	0.462 (0.111–1.917) ^&^ 0.412 (0.080–2.118)	0.288 0.288	0.466 (0.112–1.943) ^&^ 0.297 (0.040–2.190)	0.294 0.234
	*χ*^2^ = 0.974; *p* = 0.324									
C	31	0.456	9	0.321	1.769 (0.701–4.463) ^&^ 1.872 (0.689–5.086)	0.227 0.219	1.994 (0.698–5.697) ^&^ 2.097 (0.665–6.610)	0.198 0.206	2.039 (0.707–5.877) ^&^ 2.312 (0.635–8.417)	0.187 0.204
T	37	0.544	19	0.679	0.565 (0.224–1.427) ^&^ 0.540 (0.196–1.484)	0.227 0.232	0.502 (0.176–1.433) ^&^ 0.483 (0.158–1.471)	0.198 0.200	0.490 (0.170–1.414) ^&^ 0.445 (0.132–1.503)	0.187 0.192
	*MTHFR* c.1286A>C									
c.[1286A=];[1286A=]	3	0.088	3	0.214	0.355 (0.062–2.025) ^&^ 0.342 (0.050–2.328)	0.244 0.273	0.460 (0.062–3.411) ^&^ 0.431 (0.039–4.729)	0.448 0.491	0.491 (0.067–3.602) 0.338 (0.009–1.287	0.484 0.559
c.[1286A>C];[1286A=]	12	0.353	9	0.643	0.303 (0.083–1.112) ^&^ 0.287 (0.072–1.150)	0.072 0.078	**0.081 (0.011–0.576)** **^&^ 0.052 (0.005–0.580)**	**0.012** **0.016**	**0.073 (0.010–0.548)** **^&^ 0.039 (0.002–0.687)**	**0.011** **0.027**
c.[1286A>C];[1286A>C]	19	0.559	2	0.143	**7.600 (1.470–39.274)** **^&^ 7.355 (1.322–40.930)**	**0.016** **0.023**	**24.652 (2.024–257.429)** **^&^ 25.633 (2.222–295.696)**	**0.007** **0.009**	**25.883 (2.430–275.707)** **40.327 (1.860–874.464)**	**0.007** **0.019**
	*χ*^2^ = 5.986; *p* < 0.015									
A	18	0.265	15	0.536	**0.312 (0.125–0.781)** **^&^ 0.303 (0.117–0.784)**	**0.013** **0.014**	**0.238 (0.082—0.691)** **^&^ 0.218 (0.062–0.772)**	**0.008** **0.018**	**0.239 (0.082–0.700)** **^&^ 0.209 (0.055–0.793)**	**0.009** **0.021**
C	50	0.735	13	0.464	**3.205 (1.280–8.023)** **^&^ 3.354 (1.319–8.527)**	**0.013** **0.011**	**4.207 (1.447–12.229)** **^&^ 4.437 (1.356–14.523)**	**0.008** **0.014**	**4.176 (1.429–12.202** **^&^ 4.930 (1.403–17.321)**	**0.009** **0.009**

OR, presented as OR (95% CI—+95% CI), calculated with the aid of multiple logistic regression analysis; crude OR values are adjusted for age and sex (*) or for age, sex, serum folic acid, and serum 5-MTHF (^#^). *p* < 0.05, along with corresponding ORs, are in bold. ^&^ the bootstrap-boosted OR values, estimated with the classical resampling procedure with 10,000 iterations. For χ^2^ estimates, the *p* values were calculated with the Yates-corrected Chi-square test or Fisher’s exact test.

**Table 3:** Serum concentrations of folates (folic acid, 5-MTHF) in carriers of various *MTHFR* polymorphisms.

Genotype	*n*	Folic Acid	*p*	5-MTHF	*p*
*MTHFR* c.665C>T					
c.[665C=];[665C=]	14	1.95 (1.625–2.075) ^#,&^		3.65 (3.350–3.875) ^#,&^	
c.[665C>T];[665C=]	12	1.90 (1.700–2.125) ^&^	0.414 ^&^	3.80 (3.475–3.925) ^&^	0.357 ^&^
c.[665C>T];[665C>T]	22	1.90 (1.825–2.100) ^#^	0.364 ^#^	3.75 (3.525–3.900) ^#^	0.347 ^#^
*MTHFR* c.1286A>C					
c.[1286A=];[1286A=]	6	1.80 (1.650–2.025) ^#,&^		3.70 (3.700–3.775) ^#,&^	
c.[1286A>C];[1286A=]	21	1.90 (1.600–2.100) ^&^	0.505 ^&^	3.70 (3.000–3.900) ^&^	0.448 ^&^
c.[1286A>C];[1286A>C]	21	2.00 (1.900–2.100) ^#^	0.181 ^#^	3.80 (3.500–3.900) ^#^	0.428 ^#^

Data, given in μg/L, are presented as median and quartile ranges. Values determined with the use of LC-MS. The significance of differences between the groups was assessed using the bootstrap-boosted Kruskal–Wallis test. ^#^, ^&^—compared to the group 1. Abbreviations: LC-MS (Liquid Chromatography-Mass Spectrometry).

**Table 4:** Mean serum folates (folic acid, 5-MTHF) concentrations according to the presence of coronary artery disease and *MTHFR* genotype.

Genotype	Folic Acid					5-MTHF				
	CAD^+^	*n*	CAD^−^	*n*	*p*	CAD+	*n*	CAD^−^	*n*	*p*
*MTHFR* c.665C>T										
c.[665C=];[665C=]	1.9 (1.6–2.05)	11	2.0 (1.8–3.65)	3	0.316	3.5 (3.1–3.8)	11	3.9 (3.8–4.15)	3	0.079
c.[665C>T];[665C=]	2.0 (1.7–2.5)	9	1.8 (1.55–1.9)	3	0.205	3.9 (3.6–3.9)	9	3.7 (2.8–3.9)	3	0.391
c.[665C>T];[665C>T]	1.9 (1.825–2.1)	14	1.95 (1.75–2.1)	8	0.454	3.7 (3.525–3.9)	14	3.8 (3.525–3.9)	8	0.427
*MTHFR* c.1286A>C										
c.[1286A=];[1286A=]	1.8 (1.55–4.5)	3	1.8 (1.7–1.95)	3	0.4	3.7 (3.1–4.25)	3	3.7 (3.7–3.75)	3	0.5
c.[1286A>C];[1286A=]	1.8 (1.675–2.05)	12	2.0 (1.3–2.1)	9	0.357	3.65 (3.2–3.9)	12	3.9 (3.0–4.0)	9	0.269
c.[1286A>C];[1286A>C]	2.0 (1.85–2.1)	19	1.95 (1.925–1.975)	2	0.433	3.7 (3.5–3.9)	19	3.85 (3.825–3.875)	2	0.286

Data are presented as median and quartiles. The concentrations, given in μg/L, were determined using LC-MS (for details, see ‘”Section 2”). The significance of differences between the groups was assessed using the bootstrap-boosted Mann–Whitney *U* test. Abbreviations: 5-MTHF, 5-methylenetetrahydrofolate; CAD, coronary artery disease; LC-MS, liquid chromatography mass spectrometry); SD (standard deviation).

## Data Availability

The data presented in this study are available on request from the corresponding author. The data are not publicly available due to privacy.
